# Rectal stimulation with prebiotics and probiotics before ileostomy reversal: a study protocol for a randomized controlled trial

**DOI:** 10.1186/s13063-023-07065-x

**Published:** 2023-01-17

**Authors:** Marília Marcelino, Francisco Tustumi, Lucas Soares Gerbasi, Rafael Vaz Pandini, Rafaela de Souza Novo, Marleny Novaes Figueiredo de Araujo, Elis Oliveira, Victor Edmond Seid, Sergio Eduardo Alonso Araujo

**Affiliations:** Department of Coloproctology, Hospital Israelita Albert Einstein, Av. Albert Einstein, 627 - Jardim Leonor, São Paulo, SP 05652-900 Brazil

**Keywords:** Colorectal neoplasms, Colorectal surgery, Ileostomy, Rectal neoplasms, Rectum, Proctocolectomy

## Abstract

**Background:**

Ileostomy closure is associated with a high rate of postoperative morbidity, and adynamic ileus is the most common complication, with an incidence of up to 32%. This complication is associated with delayed initiation of oral diet intake, abdominal distention, prolonged hospital stay, and more significant patient discomfort. The present study aims to evaluate the rectal stimulus with prebiotics and probiotics before ileostomy reversal.

**Methods:**

This is a protocol study for an open-label randomized controlled clinical trial. Ethical approval was received (CAAE: 56551722.6.0000.0071). The following criteria will be used for inclusion: adult patients with rectal cancer stages cT3/4Nx or cTxN+ that underwent loop protection ileostomy, patients treated with neoadjuvant chemoradiotherapy, and patients who underwent laparoscopic or robotic total mesorectal excision. Patients will be randomized to one of two groups. The intervention group (with rectal stimulus): the patients will apply 500 ml of saline solution with 6 g of Simbioflora® rectally, once a day, for 15 days before ileostomy closure. The control group (without rectal stimulation): the patients will close the ileostomy with no previous rectal stimulus. The primary outcomes will be the adynamic ileus (need for postoperative nasogastric tube insertion; nausea/vomiting; or intolerance to oral feedings within the first 72 h) and intestinal transit (time to first evacuation/flatus).

**Results:**

The patient’s enrollment starts in January 2023. We expect to finish in July 2025.

**Discussion:**

The findings of this randomized clinical study may have important implications for managing patients undergoing ileostomy reversal.

**Trial registration:**

This study is registered in the Brazilian Trial Registry (ReBEC) under RBR-366n64w. Registration date: 19/07/2022

## Background

A protective ileostomy is a temporary measure usually applied to reduce the severity of complications related to colorectal anastomosis in patients with rectal cancer undergoing neoadjuvant chemoradiotherapy [1]. However, ileostomy closure is associated with a high morbidity rate, and adynamic ileus is the most common complication, with an incidence of up to 32% [2–12]. This complication is associated with delayed initiation of oral diet intake, abdominal distention, more significant patient discomfort, and prolonged hospital stay [13]. The probable cause is colonic dysfunction, leading to mucosal atrophy, with the consequent loss of intestinal absorptive capacity and atrophy of intestinal muscles, inducing changes in intestinal peristalsis [14].

Abrisqueta et al. [12], in a clinical trial of 70 patients, showed that patients that stimulated the efferent ileostomy loop with 500 ml of saline plus a thicker agent (Nestlé Resource, Vevey, Switzerland) 2 weeks before ileostomy closure had a shorter time for diet acceptance (1.06 *vs.* 2.57 days), shorter mean time to defecation (1.14 *vs.* 2.85 days), lower rate of adynamic ileus (3% *vs.* 20%), and shorter hospital stay (2.5 *vs.* 4.6 days). However, the study encompassed a limited sample size, including both primary open and laparoscopic surgery. Open primary surgery can falsify the diagnosis of adynamic ileus since intestinal adhesions can contribute to postoperative food intolerance [15].

Rodríguez-Padilla et al. [16], in a recently published randomized clinical trial, evaluated 69 patients. The intervention group had the efferent ileostomy loop stimulated with probiotics (Vivomixx®). The study demonstrated a decrease in the severity of colitis. However, there was no clinical implication for this finding (the groups showed similarities in the length of hospital stay, adynamic ileus, and time to the first defecation). It is noteworthy that Rodríguez-Padilla et al. [16] performed a per-protocol (not intention-to-treat) analysis, and the primary outcome was colitis rate. The sample size was estimated based on the presumed difference in colitis rates between the groups, which does not necessarily imply clinical repercussions. Thus, the differences between the groups for the length of stay and time for diet acceptance probably did not present enough power (1-beta) to conclude a non-significant clinical relevance for intestinal stimulation. Per-protocol analyzes are weak for clinical practice inferences, especially in a study protocol in which the intervention depends on the patient's active participation (which can be erroneous) to infuse the substances that will stimulate the efferent loop.

In an observational study, Liu et al. [17] evaluated outpatients that self-administered reinfusion with *succus entericus* prior to ileostomy closure. The group that received *succus entericus* showed a significantly shorter time to first flatus or stool (27.9 *vs.* 32.3 h), shorter length of hospital stay (4.9 *vs.* 5.52), and better low anterior resection scores (LARS).

These previous studies evaluated the excluded intestinal stimulus via the efferent ileostomy loop. However, self-delivering substances through ileostomy are not usual for outpatients, who may fail to stimulate excluded intestinal loop appropriately, creating complexity in interpreting the results. The stimulation of the rectum was not evaluated in these previous trials. Rectal enemas are often used and tolerated as routine treatment for several conditions, such as inflammatory bowel disease and chronic constipation [18–21]. Consequently, theoretically, enemas could be easier for patients to adhere to an excluded intestinal stimulus protocol.

We hypothesized that a rectal administration of prebiotics and probiotics may significantly impact colitis amelioration and could be well tolerated. Besides, since the rectum and colon work as a reservoir [22], their direct stimulus to the mucosal could theoretically improve clinical outcomes.

Considering the heterogenic findings among previously published trials regarding the value of intestinal stimulus with prebiotics and probiotics and the lack of a clinical study evaluating direct stimulation by the rectum, a high-quality randomized trial is needed.

### Objectives

The present study is a protocol for a 1:1 parallel-randomized superiority clinical trial that aims to evaluate, with an intention-to-treat analysis, the value of rectal stimulation with prebiotics and probiotics before ileostomy closure. We hypothesized that rectal stimulation with prebiotics and probiotics before ileostomy closure could reduce the incidence of adynamic ileus and improve patients’ postoperative clinical and functional outcomes. The findings of this study may have important implications for the future management of patients undergoing ileostomy reversal.

## Methods

**Fig. 1:**
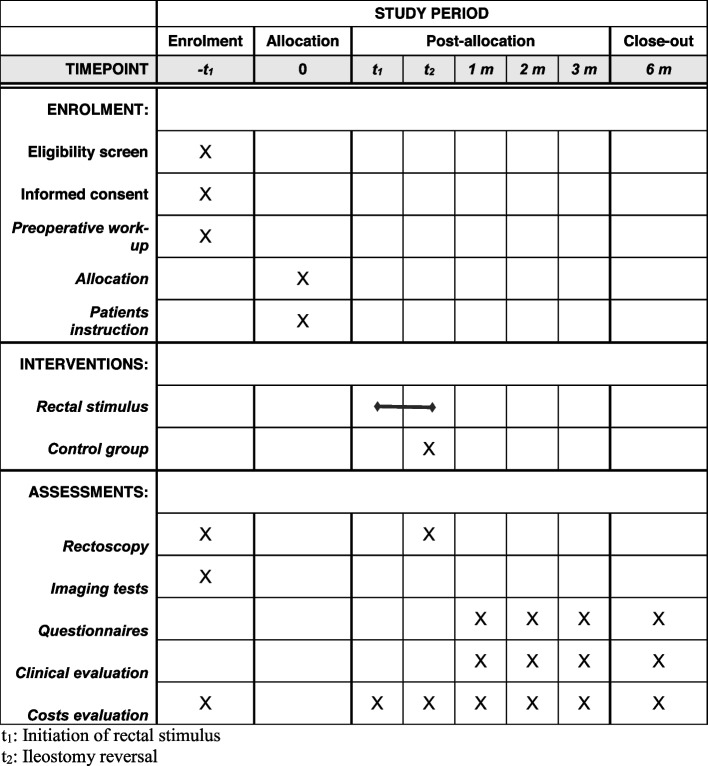
SPIRIT. Schedule of enrolment, interventions, and assessments

The SPIRIT 2013 [23] guidelines were followed for the construction of this report (Fig. 1).

### Study design

This study will be an open-label parallel-randomized superiority controlled clinical trial.

### Study settings

Patients will be recruited from a public oncological surgery teaching hospital, and all procedures will be performed in this same hospital (Hospital Municipal Vila Santa Catarina, São Paulo, Brazil).

### Ethics and registration

The study’s protocol followed the Helsinki statements and was approved by the local ethics committees (Hospital Israelita Albert Einstein; CAAE: 56551722.6.0000.0071; CEP 5.475.035). The protocol was registered in the Brazilian Registry of Clinical Trials (ReBEC) under the identifier RBR-366n64w. All participants will sign the Free and Informed Consent Form.

### Eligibility

The inclusion criteria are patients that (1) are adults (> 18 years), (2) with rectal cancer in clinical stages cT3/4Nx or cTxN+, (3) treated with neoadjuvant chemoradiotherapy, (4) submitted to laparoscopic or robotic total mesorectal excision and submitted to loop protection ileostomy, and (5) who are not undergoing systemic chemotherapy.

The exclusion criteria are patients that (1) refuse to participate in the study; (2) with a history or suspicion of inflammatory bowel disease (typical endoscopic findings at diagnosis, including marked erythema, loss of vascular marking, erosions, ulcers, and spontaneous bleeding); (3) pregnant; or (4) who have any contraindication for general anesthesia or surgical intervention.

All patients from our institution will be evaluated consecutively, and the ones that fit the eligibility criteria will be invited to participate in the study. To be transparent during the selection process, we will document every patient that does not accept to participate.

### Sample size

Assuming that the intervention will reduce the adynamic ileus incidence after ileostomy reversal from 20 to 2.8% [12], the estimated sample is 52 patients in each study arm, considering alpha (two-sided) 0.05 and beta 0.2 [24]. Considering that our primary outcome will be a short-term endpoint, evaluated during hospitalization after surgery, we do not expect a significant loss of follow-up for the primary outcome.

### Randomization

Patients will be consecutively evaluated, and the eligible patients will be invited to participate in the research project. If the patient agrees to participate in the project, the researchers will instruct about the risks and benefits of the study and will be given the printed Free and Informed Consent Term for signature. The patients will be advised that they may interrupt their participation in the research at any time. One copy will remain with the patient and the other with the research team.

The researchers will use Microsoft Excel’s random number generation function to perform a 1:1 block randomization. Blocking will ensure a close balance of the numbers in each group at any time during the trial. Knowing that investigators could deduce some of the subsequent treatment allocations if he or she knows the block size, we will use large block sizes (8–12) and randomly vary the block size to ameliorate this issue. The randomization sequence was not concealed. There is no plan for interim analysis.

**Fig. 2:**
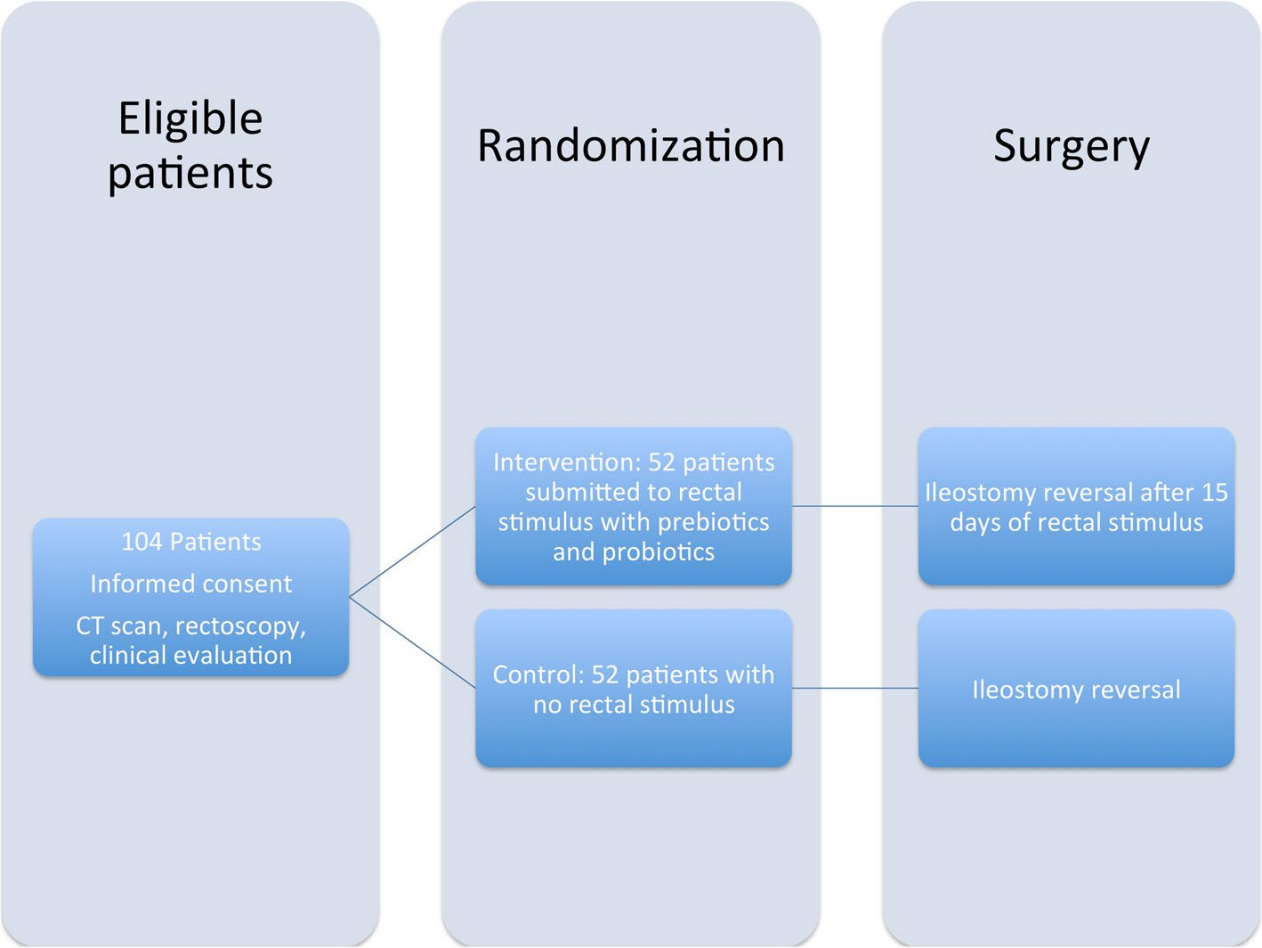
Allocation flow diagram

Patients will be randomly assigned to the intervention group (with rectal stimulus) or the control group (without rectal stimulus). See Fig. 2.

### Study groups

Intervention group (with rectal stimulation): The patient will receive guided instruction in an outpatient setting. The patient will receive the necessary materials, which include 14-French rectal tubes, saline, and a source of soluble prebiotic fibers (fructooligosaccharide) with probiotics (*Lactobacillus acidophilus*, *Lactobacillus rhamnosus*, *Lactobacillus para casei*, *Bifidobacterium lactis*) (Simbioflora®; Brand: Invictus; Manufacturer: Farmoquimica; MS1666370001001 Registry). The patient will apply 500 ml of saline solution with 6 g of Simbioflora® rectally, once daily, for 15 days before the ileostomy reversal surgery. The saline solution vials will have a predetermined volume (500 ml) to guarantee that the patients use the correct volume. The patient will have weekly visits, and the researchers will provide a contact for service at any time to optimize patients’ adherence.

Control group (without rectal stimulus): The patient will be submitted to the ileostomy reversal with no previous rectal stimulus. No placebo will be applied.

In both groups, patients will be instructed to record colic/abdominal pain symptoms, nausea/vomiting, diarrhea, and gas during the 15 days prior to ileostomy closure.

### Preoperative workup

Before surgery (1 to 3 months prior to surgery), the patients will have a thoracic and abdominal computed tomography (CT) scan and pelvic MRI for routine oncologic evaluation. CT scan will be performed with endovenous and rectal iodinated contrast. In addition, a digital examination and rectoscopy will be performed. Rectoscopy will assess the grade of colitis (Harig scoring system) [25]. During rectoscopy, if there are changes in the mucosa or anastomosis, such as inflammatory areas or suspicious malignancy, biopsies will be performed according to the usual care of all patients with rectal cancer during oncological follow-up. These procedures are already performed for the usual routine service management before ileostomy closure.

All patients will then undergo a standardized preoperative clinical and anesthesiological evaluation.

### Surgical techniques and surgeons

Five experienced surgeons will perform the procedures (all performed more than 50 ileostomy closures).

Patients will have 8 h of fasting. Infusion of second-generation cephalosporins will be performed intravenously during anesthetic induction before the procedures.

The initial approach (total mesorectal excision with protective ileostomy) will be performed with minimally invasive access (robotic-assisted or laparoscopic) following the following technique:

1—Patient in lithotomy position under spinal anesthesia and sedation; 2—Antisepsis with alcoholic chlorhexidine solution; 3—Asepsis and placement of sterile surgical drapes; 4—Inspection of the cavity for the presence of metastases; 5—Identification, isolation, and sealing of the inferior mesenteric vein at its origin; 6—Left mesocolon detachment from retroperitoneum structures; 7—Access to the retrocavity of the epiplons, and opening of the intercolonepiploic space, with release of the splenic angle; 8—Identification, isolation, and clipping of the inferior mesenteric artery at its origin; 9—Release of left parietocolic gutter; 10—Mesorectal dissection up to the level of the levator ani muscles and posterior section of the rectum; 11—Colorectal anastomosis with Circular Stapler (CDH 29); 12—Construction of a protective ileostomy 30 cm from the ileocecal valve, in loop, with its fixation through a hole created in the right hemiabdomen, through the rectus abdominis muscle, and fixation with Vycryl 3-0; 13—Review of hemostasis; synthesis of aponeurosis and skin; 14—Dressings.

The second surgery (ileostomy reversal) will be performed at least two months after the first surgery, after adjuvant therapy (when indicated), and the following technique will be followed:

1—Patient in lithotomy position under spinal anesthesia and sedation; 2—Antisepsis with alcoholic chlorhexidine solution; 3—Asepsis and placement of sterile surgical drapes; 3—Circular incision around the ileostomy, opening in layers until the release of the ileostomy from the aponeurosis; 4—Barcelona technique anastomosis; 5—Review of hemostasis; synthesis of aponeurosis and skin; 6—Dressings.

A rectoscopy will be performed intraoperatively to assess colitis grade (Harig scoring system) [25].

### Postoperative follow-up

In both surgeries, the patient will be discharged following the Enhanced Recovery After Surgery (ERAS®) protocol [26] when there is no evidence of complications, full diet acceptance, and bowel movement or flatus. The patient will be offered a liquid diet in the immediate postoperative period (4–6 h after the surgery) and a clear liquid diet after 24 h of the surgery if the patients present no nausea or abdominal distension. The diet will consist of foods that are low in fiber and fat. Postoperatively, patients will be instructed to record symptoms of colic/abdominal pain, nausea/vomiting, diarrhea, and the general appearance of stools and gas.

The patients will be followed at least 6 months after the surgery. Patients will return after 7–14 days after surgery, followed by 1, 3 months, and 6 months. At each return visit, patients will be actively investigated and questioned regarding food acceptance, stool consistency, signs of fecal incontinence, abdominal cramping/pain, bowel movements per day, and quality of life. The relevant questionnaires will be applied at each follow-up visit.

Patients will undergo the usual tests for colorectal cancer restaging, including serum laboratory tests, CT scans, MRI, and rectoscopy.

### Outcomes

The primary outcome will be as follows: (1) incidence of adynamic ileus (the number of patients needing postoperative nasogastric tube insertion; nausea/vomiting; or intolerance to oral feedings in the first 72 h of the surgery).

Secondary outcomes will be as follows: (1) the time to intestinal transit (time to first evacuation/flatus); (2) time to start feeding; (3) length of hospital stay; (4) grade of colitis (Harig scoring system) [25]; (5) operative complications (during hospitalization); (6) direct costs; (7) stool consistency (Bristol stool scale) [27]; (8) fecal incontinence (Wexner score) [28]; (9) abdominal cramps/pain; (10) number of evacuations per day; and (11) quality of life (SF-36) [29] and low anterior resection syndrome score (LARS) [30]. The secondary outcomes 7 to 11 will be evaluated at every clinical visit during postoperative follow-up. Patients will return after 7–14 days after surgery, followed by 1, 3 months, and 6 months. Patients will be actively investigated for secondary outcomes at each return visit. The relevant questionnaires will be applied at each follow-up visit.

### Cost assessment

The direct medical costs of treatment will be described from the institution's perspective. The objective of this cost analysis is to estimate the additional costs related to the intervention and if these additional costs will be overcome by hypothetical cost reduction due to a reduction in the length of hospital stay or other costs related to patient care.

A mixed methodology of micro and macro-costing will be used. Costs related to hospitalization and surgery (operating room time, medical and multidisciplinary consultations, length of hospital stay, ICU stay, and outpatient consultations) will be evaluated. Fixed and variable costs (human resources, material resources, and infrastructure) will be estimated by evaluating the respective unitary values of the institution’s costs. Medicines, medical devices, nutrition, blood, laboratory, and imaging studies will be evaluated by calculating the microcost according to the individual consumption of the patient multiplied by the respective acquisition cost. Operating room time will be measured from patient entry to operating room exit, including anesthesia and surgery. Costs will be presented in the Brazilian Real (R$) (R$ 1.00 = US$0.19; July 31, 2022).

### Institutional resources and infrastructure

The Municipal Hospital Vila Santa Catarina, managed by Einstein in partnership with the public Brazilian health system (SUS), offers all the human and material resources necessary for the project. The hospital has a specialized coloproctology team with operating rooms for the procedures proposed for this project.

### Statistical analysis

An independent author not involved in the recruitment or data collection will perform an intention-to-treat analysis in this superiority controlled trial. Improper application of the rectal tubes will not be excluded from the analysis. The patients will be analyzed according to the group assigned in randomization, even if they drop the intervention or apply only partially (or incorrectly) the rectal stimulation solution. Qualitative variables will be described as absolute counts and percentages. Continuous variables will be described as median and interquartile range. Scores outcomes generated from questionnaires will be evaluated as discrete variables. Differences between groups will be evaluated by the Mann-Whitney test for continuous and discrete variables and Fisher’s exact test or chi-square test for categorical variables. No adjustments per baseline characteristics of the patients are intended.

The investigators will check monthly to ensure the integrity of the data quality, assessing for missing data and invalid field entries. The mean of each variable will be used to replace missing data. The Kolmogorov-Smirnov test will be used to investigate the normality distribution of the variables. For non-normal distribution, a logarithmic transformation will be performed. For sensitivity analysis, the best-worst-case scenario dataset will be generated, assuming all participants lost to follow-up in the intervention group have had a beneficial outcome and all those with missing outcomes in the control group have had a harmful outcome. Then a worst-best-case scenario dataset will be generated assuming all participants lost to follow-up in the intervention group have had a harmful outcome, and all those lost to follow-up in control have had a beneficial outcome. For continuous outcomes, a “beneficial outcome” will be the group mean plus 2 standard deviations of the group mean, and a “harmful outcome” will be the group mean minus 2.

A per-protocol analysis, in which participants who violate the protocol are excluded from the analysis, will be performed as sensitivity analysis to investigate the robustness of the results to protocol deviations.

Statistical analyzes will be performed using the Statistical Package for the Social Sciences, version 18.0 for Windows (SPSS Inc., Chicago, IL, USA).

### Risks and inconveniences

The additional actions performed to fulfill the protocol comprise the rectal stimulation in the interventional group, the weekly visits while administering rectal stimulation, and the rectoscopy prior to surgery. The remaining procedures and visits are performed according to the usual clinical practice. The postoperative follow-up will be the same for intervention and control groups.

No prior information suggests the risk for serious adverse events due to the trial’s intervention. The main risks associated with the procedures are colic or abdominal pain, nausea or vomiting, diarrhea, and gas. If the participant has symptoms, they will receive the corresponding medications to treat them. The research team will provide a contact for service at any time for guidance and control of symptoms related to the procedures. In the case of any serious adverse events related to the intervention, our institution and research staff will provide all the necessary care. A data safety committee will not be established for this study since this is a low-risk trial. This study will not be performed in patients with an elevated risk of death or serious adverse events. All patients will be preoperatively evaluated with standard clinical and anesthetic, and pregnant patients or patients who have any contraindication for general anesthesia or surgical intervention will be excluded (see eligibility criteria above).

Exam data are encrypted in a specific medical record system at Hospital Israelita Albert Einstein, ensuring that data confidentiality is not compromised.

### Data monitoring and management

The researchers will perform data collection. The data will be stored in a secured database using protected pathways. All data is anonymous. Only the research team will have access to the data set.

Independently on the findings of the present study, we intend to publish it in a peer-review journal. The local ethics committee does not require a data monitoring committee since only low-risk interventions will be performed. However, an audition may be performed at the discretion. No interim analyses are planned. The only stopping guideline is when the planned sample size is reached.

### Strategies to ensure data privacy

The study will follow the necessary precautions for the Brazilian General Data Protection Law (*Lei Geral de Proteção de Dados Pessoais – LGPD*; Amends Law #13,709). A data anonymization technique will be applied. The researchers will exclude information that may lead to identifying the data subject. All anonymized data will be kept under the responsibility of the coordinating researchers in an Excel file (Microsoft®) protected with a password. The researchers commit not to share the data.

### Study benefit

Suppose there is evidence of the effectiveness of probiotics and prebiotics. In that case, the patients selected for the intervention group will have received treatment to avoid postoperative symptoms, such as food intolerance.

The findings of this study may have important implications for the management of patients undergoing ileostomy reversal.

### Protocol amendments

Any amendments to the protocol will be included in the trial registry once the local ethics committee approves. The results of this trial will be published in peer-reviewed journals.

## Discussion

Adynamic ileus is a frequent postoperative complication after ileostomy reversal. Preventing this complication and improving functionality may impact the quality of life-related to bowel movements and reduce hospitalization and costs. Consequently, the findings of this randomized clinical study may have important implications for the management of patients undergoing ileostomy reversal.

Previous trials [12, 16, 31] proposed stimulus of the excluded colon via efferent ileostomy loop. This is the first controlled trial for rectal stimulus protocol with prebiotics and probiotics. The application of any intestinal stimulus by the patient demands high patient collaboration, which frequently is not easy. Patients may fail to apply the probiotics via rectal tubes properly. Thus, a per-protocol analysis would not give the most accurate depiction of the exact clinical practice applicability. The intention-to-treat analysis, as proposed for this protocol, shall provide a high level of evidence for the use of this intervention in clinical practice. The current trial may give a clear depiction of the applicability of intestinal stimulus prior to ileostomy reversal in the real world.

As a limitation, this trial will evaluate 15 days of rectal stimulation. Future studies will still be needed to determine the exact stimulation period to guarantee the rectal stimulus's highest efficacy. Other limitations include that this trial is a monocentric and unblinded study. Monocentric studies may impose some loss in external validity. However, the findings of this study may encourage the use of rectal stimulation in other institutions, and future multicentric randomized trials may contribute to the generalization of the findings of our study.

## Trial status

This is version 1 of the protocol (registration on 19 July 2022). The patient’s enrollment starts in January 2023. Considering the surgical volume in the last 3 years in our institution (25–50 ileostomy reversal procedures), we expect to finish in 2025. We intent to extend the enrolment period if necessary, until the completion of the study.

## Data Availability

No datasets were generated or analyzed during the current study. All relevant data from this study will be made available upon study completion.
